# Femtosecond Laser-Induced Periodic Surface Structures on Different Tilted Metal Surfaces

**DOI:** 10.3390/nano10122540

**Published:** 2020-12-17

**Authors:** Yi-Hsien Liu, Kong-Kai Kuo, Chung-Wei Cheng

**Affiliations:** Department of Mechanical Engineering, National Chiao Tung University, No. 1001 Ta Hsueh Road, Hsinchu 300, Taiwan; kevin820624@gmail.com (Y.-H.L.); kevinkuo031@gmail.com (K.-K.K.)

**Keywords:** femtosecond laser, laser-induced periodic surface structures, LIPSS, LSFL

## Abstract

Laser-induced periodic surface structures (LIPSS) are used for the precision surface treatment of 3D components. However, with LIPSS, the non-normal incident angle between the irradiated laser beam and the specimen surface occurs. This study investigated LIPSS on four different metals (SUS 304, Ti, Al, and Cu), processed on a tilted surface by an s-polarized femtosecond fiber laser. A rotated low spatial frequency LIPSS (LSFL) was obtained on SUS 304 and Ti materials by the line scanning process. However, LSFL on Cu and Al materials was still perpendicular to the laser polarization. The reason for the rotated and un-rotated LSFL on tilted metal surfaces was presented. The electron-phonon coupling factor and thermal conductivity properties might induce rotational LSFL on tilted SUS 304 and Ti surfaces. When fabricating LSFL on an inclined plane, a calibration model between the LSFL orientation and inclined plane angle must be established. Hence, the laser polarization direction must be controlled to obtain suitable LSFL characteristics on a 3D surface.

## 1. Introduction

LIPSS (Laser-induced periodic surface structures) were first discovered by Birnbaum in 1965 [1] and gradually extended from basic research to application [2,3,4]. They can be quickly generated on metal, semiconductor, and dielectrics by a single laser beam [5]. In the past, most papers focused on generating LIPSS on plant surfaces by different scanning strategies or polarization [6,7,8,9]. Recent studies recently investigated LIPSS by the p-polarized laser beam with oblique incidence on the sample surface [10,11,12,13]. These studies found that the polarization portion irradiated on the hole and groove sidewall changes with the incidence angle. The low spatial frequency LIPSS (LSFL) on the sidewall changes direction, for instance, not being perpendicular to the polarization direction. However, few studies investigated LIPSS by the s-polarized laser beam with oblique incidence on the sample surface. The studies showed that in this condition, the laser polarization portion on the specimen surface will not change.

Recently, Schwarz et al. used a scanning s-polarized femtosecond laser to irradiate materials (e.g., fused silica) with a tilted angle [14]. The study found that the LSFL orientation was not perpendicular to the laser polarization, and the rotation angle increased as the specimen tilted angle increased. In another study, Liu et al. [15] investigated the LIPSS generated by linear polarization (s-polarized and p-polarized) femtosecond laser beam on a tilted SUS 304 surface. The experiments found that LSFL was rotated by the s-polarized beam and was not rotated by the p-polarized beam. Hence, the mechanism of rotational LSFL on the tilted specimen by s-polarized laser beam was deduced. However, although [14,15] have published the experimental results for rotational LIPSS on different materials by s-polarized femtosecond laser beam, there is no study to validate whether the LSFL rotates on the tilted surface for more metal materials.

In this study, LIPSS was generated on four metals (SUS 304, Ti, Al, and Cu) by an s-polarized femtosecond laser beam (1030 nm) with specimen tilted angles (0°, 30°, and 45°) and validated whether the LSFL rotated or not. These metal materials were chosen because they have different optical and thermophysical properties and are suitable to investigate the mechanism of rotational LSFL. The results reveal that only specific metals at the processing wavelength can exhibit rotational LSFL structures on the tilted surface.

## 2. Materials and Methods

The mechanically polished materials (SUS 304, Ti, Al, and Cu) were irradiated using a femtosecond fiber laser (KASMORO, mRadian Inc, Hsinchu, Taiwan) with a wavelength of 1030 nm, a pulse width of 420 fs, a repetition rate of 100 kHz, and a maximum laser power of 2 W. The laser beam was linearly polarized and was passed through a liquid lens (EL-10-42-OF, Optotune Inc., Dietikon, Switzerland) before entering a galvanometric scanner that had an F-theta lens with a focal length of 100 mm. The liquid lens was used to control the focus position on the tilted surface. The focused spot size was determined to be 60 µm. It is important to note that the experiment was mainly based on the wavelength of 1030 nm, but for the copper material, to explore the influence of the laser wavelength, the laser beam was passed through the BBO crystal to convert the laser beam with wavelength 515 nm. The laser beam was entering a galvanometric scanner that had an F-theta lens with a focal length of 165 mm. The focused spot size was determined to be 50 µm.

**Figure 1:**
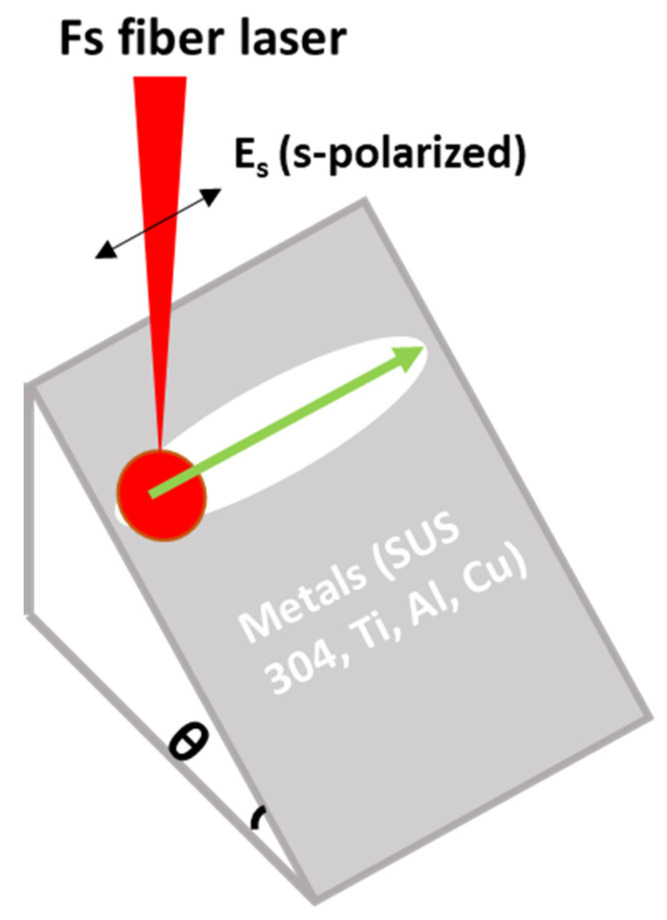
Schematic of fabricating the low spatial frequency LIPSS (LSFL) on different metals.

The experiment process is shown in Figure 1. The line scanning with the s-polarized beam parallel to the scanning direction was used to investigate LSFL on four metals (SUS 304, Ti, Al, and Cu) with different sample tilted angles (θ). For the femtosecond laser processing, all the specimens were cleaned in an ultrasonic bath (in water) machine for 10 min, and the experiment was conducted in an airy atmosphere. After the experiment, scanning electron microscopy (SEM, Hitachi SU-8010, Tokyo, Japan) was used to measure the structure profile. The orientation and the period of structures were determined using a fast Fourier transformation (FFT) analysis of the SEM images.

## 3. Results and Discussion

**Figure 2:**
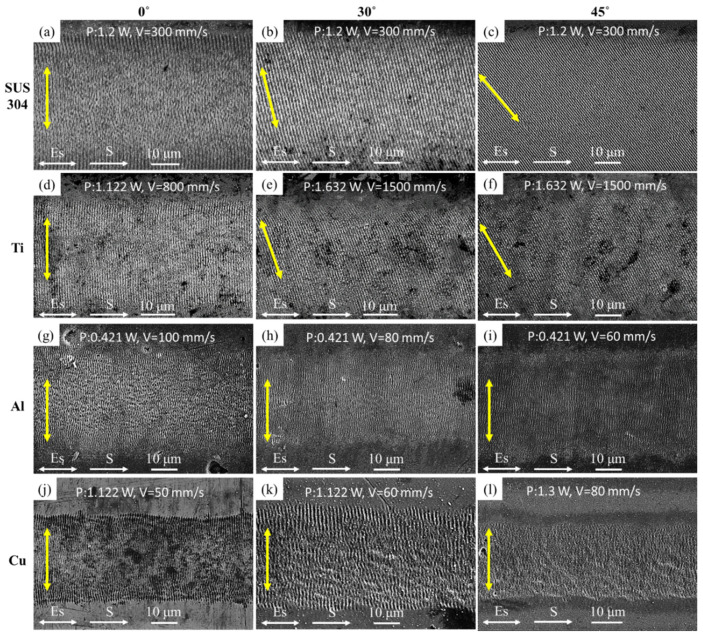
SEM images of the LSFL generated by the line scanning process for the four different metals irradiated by 1030 nm laser with different θ (0°, 30°, and 45°): (**a**–**c**) SUS 304, (**d**–**f**) Ti, (**g**–**i**) Al, and (**j**–**l**) Cu.

Figure 2 shows a line pattern with LSFL fabricated by the femtosecond laser of wavelength 1030 nm and different θ (0°, 30°, and 45°). The scanning direction is from left to right (see Figure 1). The laser power (P) and scanning speed (V) were shown on the top side of the figures. Note that these parameters were experimentally determined so that LSFL can be uniformly fabricated on different metals. Table 1 summarizes LSFL experimental results and material related optical and thermal parameters. In the θ = 0° case, as shown in Table 1, the period of LSFL was near the wavelength, that is, in the range of 907~976 nm by laser wavelength 1030 nm.

In Figure 2a,d,g,j, the orientation of LSFL (yellow double arrows) was nearly perpendicular to the laser polarization. The mechanism of generating LSFL can be explained by the interference between the incident laser beam and the surface plasmon polariton (SPP) [3]. Interestingly, at θ = 30° and 45°, as shown in Figure 2b,c,e,f, LSFL presented a different orientation for SUS 304 and Ti. When θ was increased, the LSFL rotation angle is increased. However, for Al and Cu, the LSFL is always perpendicular to the laser polarization rather than rotation, as shown in Figure 2h,i,k,l.

**Figure 3:**
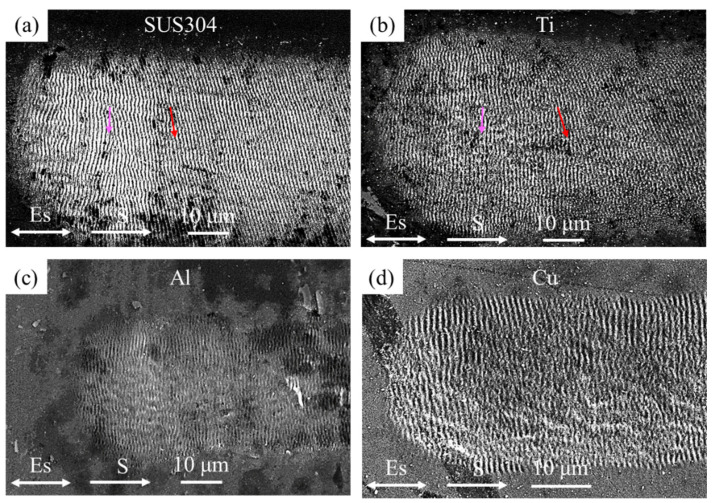
SEM images of the line scanning start regions for four materials with θ = 30°; (**a**) SUS304, (**b**) Ti, (**c**) Al, and (**d**) Cu.

Figure 3 shows the SEM images of the line scanning start region for four materials. An interesting phenomenon was observed in the scanning start region for SUS 304 and Ti, as shown in Figure 3a,b. The LSFL was perpendicular to the polarization at the center of the starting point (pink arrow) before gradually rotating along the scanning direction. It then remained at a constant rotated angle (red arrow). The LSFL rotation angle was around 14° and 19° for SUS 304 and Ti, respectively. However, in the Al and Cu material, as shown in Figure 3c,d, the LSFL was not rotated in the scanning start region. Their orientation remained perpendicular to the laser polarization.

Why is it that not all tilted metal materials can produce LSFL rotation? The possible reasons need to be further discussed. Bonse and Gräf show that the formation of LSFL may be caused by electromagnetic effects or matter reorganization [3]. This study will examine the two issues and discuss why only SUS 304 and Ti produce LSFL rotation among the four experimental materials.

Assume that SPP orientation on the tilted sample differs from the flat surface and induces the LSFL rotation. The experiment results show that the rotational LSFL only exists in SUS304 and Ti. We can deduce that the SPP generated on the tilted sample is different among the four materials. The LSFL related to SPP propagation length (Lspp) is presented in [18,19]. Recently, studies have further proposed that Lspp is related to the uniformity of LSFL. The shorter the Lspp length, the better the LSFL uniformity in metals [20]. Metals (Ti, Steel, Mo) can produce highly regular LSFL by femtosecond laser irradiation with wavelength 1030 nm. However, the LSFL were not regular for Cu and Al. The Lspp can be calculated by:

(1) Lspp=12Im(β) β=2πλε1+ε

where λ is the laser wavelength, *ε* is the relative permittivity, and β is the SPP wave number.

**Figure 4:**
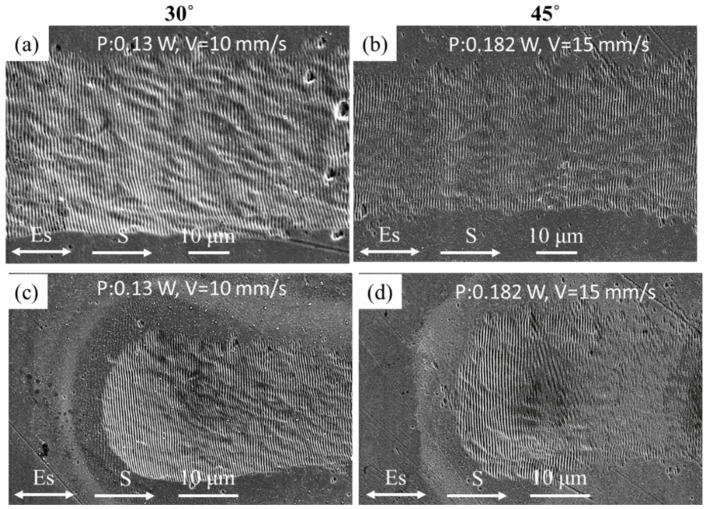
SEM images of the LSFL generated by the line scanning process for the Cu irradiated by 515 nm laser with (**a**,**c**) θ = 30° and (**b**,**d**) 45°; (**c**,**d**) scanning start regions.

The calculated Lspp at room temperature is shown in Table 1. It was found that the SUS 304 and Ti materials with shorter Lspp (<5 µm) and the Cu and Al materials with Lspp larger than 50 µm at laser wavelength 1030 nm. It will be necessary to conduct experiments with different wavelengths to validate the speculation that Lspp is a possible factor to induce LSFL rotation on a tilted surface. As shown in Table 1, the Lspp is shorter in wavelength of 515 nm (<1 µm). Therefore, the LSFL generated by a 515 nm laser was inferred to be rotated. However, as shown in Figure 4, the LSFL remains perpendicular to the laser polarization. The different wavelengths (1030 nm and 515 nm) irradiation on the Cu demonstrate that Lspp is not a critical factor to induce LSFL rotation. However, the findings validate that the uniformity of LSFL generated by 515 nm laser (Figure 4c) is better than that generated by 1030 nm laser (see Figure 3d).

**Figure 5:**
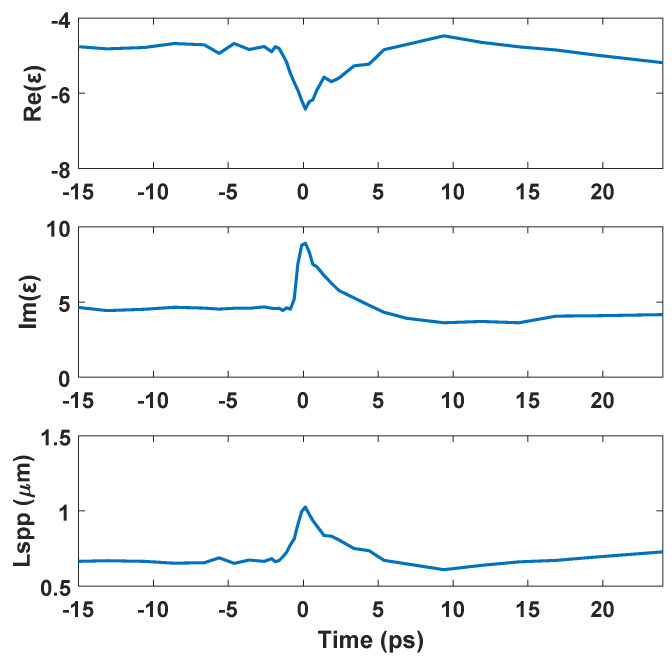
Dynamic permittivity ε and Lspp.

When the femtosecond laser was irradiated on the metal sample, the surface reflectivity was varied due to the dynamic electron temperature. We then further investigated the dynamic permittivity and Lspp to know whether they induced the rotated LSFL or not. Winter et al. used pump-probe ellipsometry to measure the dynamic relative permittivity ε when the Cu was irradiated by 528 nm laser and 0.4 J/cm^2^ [21]. The dynamic Lspp during femtosecond laser irradiation can be calculated by Equation (1) with the experimental data (dynamic ε) from [21]. As shown in Figure 5, Lspp was increased during laser irradiation. The maximum Lspp was 1.03 (at 0.14 ps) and was slightly higher than 0.78 at room temperature. However, the LSFL was still perpendicular to the laser polarization. Thus, this study speculates that Lspp was not a factor that affected LSFL rotation on the tilted sample.

We tried to compare whether the matter reorganization by material properties could influence rotational LSFL. Gurevich et al. proposed the three steps for the generation of LIPSS [22]: (1) modulation of the electron temperature ($T_{e}$) by the interference of the incident laser beam and surface plasmon wave; (2) modulation of lattice temperature ($T_{l}$); (3) hydrodynamic instabilities and reorganization of the molten material on the surface.

In this study, the electron-phonon coupling factor (*G*) and the thermal conductivity of the four materials were compared (see Table 1) in an attempt to deduce the reason for rotational LSFL. The two-temperature model (TTM) can be used to describe the electron-lattice coupling effect ($G\left(T_{e}-T_{l}\right))$ in the ultrafast laser process. If the *G* is larger and the electron thermal diffusivity is smaller, the energy that can be absorbed in the irradiated area is faster and more concentrated, providing a positive feedback loop for the amplification of the lattice temperature modulation [22,23]. The *G* values of the four materials at $T_{e}$ = 10,000 K are shown in Table 1. SUS 304 and Ti were about 6–10 times the ratio of Al and Cu. Therefore, for SUS 304 and Ti, the electron temperature coupled to the lattice temperature faster, causing a larger temperature gradient in the molten pool. In addition, SUS 304 and Ti had higher melting temperatures (1723 K and 1930 K) and lower thermal conductivity (16.2 and 22 W/m-K at 300 K), which could also make the melting pool temperature gradient larger. When the melting pool produces a larger temperature gradient, it can create a larger Marangoni force and push the liquid material to a lower temperature area [24].

The influence of the thermophysical parameters of SUS 304 and Ti when processing metals on an inclined surface can easily cause local instability on the melting pool. Accordingly, the LSFL was symmetrically rotated at both sides of the irradiation area, as shown in Figure 3a,b. According to the experimental results, it was found that when fabricating LSFL on the inclined plane, a calibration model between the LSFL orientation and inclined plane angle must be established, and the laser polarization direction must be controlled to obtain a suitable LSFL orientation.

## 4. Conclusions

This study investigated s-polarized femtosecond laser beams irradiated on four metals (SUS 304, Ti, Al, and Cu) with different sample tilted angles (θ). The experimental results of the line scanning demonstrated that: (1) only SUS304 and Ti material could induce the rotation of LSFL on the tilted sample, and the LSFL rotation angle increased as θ increased; and (2) for Al and Cu material, the LSFL orientation remained perpendicular to the laser polarization as θ increased. We deduced that the electron-phonon coupling factor and thermal conductivity were quite different on the SUS 304 and Ti and could influence the LSFL rotation on the tilted surface. The higher electron-phonon coupling factor and lower thermal conductivity of materials would cause the instability molten pool and induce the rotational LSFL on the tilted surface. Thus, for 3D surface processing, precise, controlled polarization should be conducted to generate the uniform LSFL for specific materials.

## Figures and Tables

**Table 1:** Summary of LSFL experimental results and material related optical and thermal parameters.

Material	SUS 304	Ti	Al	Cu	Cu
Wavelength λ (nm)	1030	1030	1030	1030	515
LSFL period (nm) at θ = 0°	959	976	907	919	420
LSFL period (nm) at θ = 30°	893	917	906	932	438
LSFL period (nm) at θ = 45°	788	864	814	1019	496
Optical parameters					
Re (ϵ) at 300 K [16]	−6.6275	−4.2656	−95.117	−45.761	−5.3328
Im (ϵ) at 300 K [16]	23.004	27.277	27.653	4.5744	6.1794
SPP propagation length L_spp_ (μm)	4.02	4.52	57.32	73.35	0.78
Thermal parameters					
Electron-phonon coupling factor *G* at $T_{e}$ = 10,000 K (Wm^−3^K^−1^ × 10^17^) [17]	30.04	36.74	3.62	5.29	5.29
Thermal conductivity at 300 K (W/m-K)	16.2	22	235	385	385
Melting Temperature (K)	1723	1930	933	1356	1356
